# Idea density in Japanese for the early detection of dementia based on narrative speech

**DOI:** 10.1371/journal.pone.0208418

**Published:** 2018-12-05

**Authors:** Daisaku Shibata, Kaoru Ito, Hiroyuki Nagai, Taro Okahisa, Ayae Kinoshita, Eiji Aramaki

**Affiliations:** 1 Graduate School of Science and Technology, Nara Institute of Science and Technology (NAIST), 8916–5 Takayama, Ikoma City, 630–0192, Japan; 2 Graduate School of Human and Environmental Studies, Kyoto University, Yoshida-nihonmatsu-cho, Sakyo-ku, Kyoto City, 606-8501, Japan; 3 Graduate School of Medicine, Kyoto University, 54 Kawahara-cho, Shogoin, Sakyo-ku, Kyoto City, 606–8507, Japan; Hokkaido Daigaku, JAPAN

## Abstract

**Background:**

Idea density (ID), a natural language processing–based index, was developed to aid in the detection of dementia through the analysis of English narratives. However, it has not been applied to non-English languages due to the difficulties in translating grammatical concepts. In this study, we defined rules to count ideas in Japanese narratives based on a previous study and proposed a novel method to estimate ID in Japanese text using machine translation.

**Materials:**

The study participants comprised 42 Japanese patients with dementia aged 69–98 years (mean: 84.95 years). We collected free narratives from the participants to build a speech corpus. The narratives of the patients were translated into English using three machine translation systems: Google Translate, Bing Translator, and Excite Translator. The ID in the translated text was then calculated using the Dependency-based Propositional ID (DEPID), an English ID scoring tool.

**Results:**

The maximum correlation coefficient between ID calculated using DEPID-R-ADD (a modified DEPID method to calculate ID after removing vague sentences) and the Mini-Mental State Examination score was 0.473, indicating a moderate correlation.

**Discussion:**

The results demonstrate the feasibility of machine translation-based ID measurement. We believe that the basic concept of this translation approach can be applied to other non-English languages.

## Introduction

The steady increase in life expectancy has led to severe health and sociological problems. One of the most important problems associated with longer life is the rising incidence of Alzheimer’s disease [1]. Japan’s Ministry of Health, Labour and Welfare reported that more than one out of four healthy individuals aged 65 years or older would soon be afflicted with mild cognitive impairment or dementia. The total care cost per year is estimated to exceed 10 trillion yen if these patients received medical treatment for these conditions. Unfortunately, dementia is not curable with current medical therapies, but further deterioration may be prevented if the disease is detected during its early stages. Hence, it is necessary to develop techniques and technology for the early detection of dementia.

Among the various dementia screening tools, methods based on the analysis of patient narratives have drawn much attention. Snowdon et al. found that low idea density (ID) in early life was associated with an increased risk of developing Alzheimer’s disease in late life [2]. ID, which was originally developed by Kintsch and Keenan, indicates the number of propositions (also called “ideas”) in a text or utterance [3]. The computation of ID requires experts’ annotations, and therefore involves a considerable investment of time and money. For this reason, some researchers have developed software to automatically estimate ID. For example, Brown et al. developed the Computerized Propositional ID Rater (CPIDR), a program that ascertains the ID of English language texts or utterances based on part-of-speech tags [4]. In addition, Sirts et al. developed the Dependency-based Propositional ID (DEPID) method, which can exclude repeating ideas and vague sentences [5].

The CPIDR and DEPID methods have already been applied to calculating ID in studies that classified patients into healthy controls and patients with Alzheimer’s disease or dementia. Jarrold et al. reported an accuracy of 73% in distinguishing between healthy controls and patients with Alzheimer’s disease or dementia using language features such as ID [6]. ID was also used by Roark et al. to detect mild cognitive impairment in a story re-telling dataset [7].

Although previous studies have investigated ID in patients with cognitive impairment and healthy controls that were classified based on physicians’ diagnoses, they have not–to the best of our knowledge–investigated the correlations between ID and the Mini-Mental State Examination (MMSE) score. Rather than cognitive impairment itself, ID may be more related to cognitive function such as attention and calculation ability, which can be measured using the MMSE. This study targets the relationship between ID and cognitive function with the aim of filling this knowledge gap.

Because ID has never been applied in the Japanese context, this study defines ID and investigates its validity for this language by showing the correlation between ID and cognitive function. In addition, we investigate the possibility of dementia screening using ID in Japanese narratives.

## Study participants and material

### Inclusion and exclusion criteria

The criteria used for selecting the study participants are as follows:

[Inclusion criteria]

- Elderly people over the age of 65 years diagnosed with dementia by a physician.
- Patients with mild to moderate dementia (>10 points in the MMSE).
- Patients who can answer “yes” or “no” to a question, such as “Have you ever felt lonely?”
- Speakers of the Kansai dialect (spoken in the Kansai region of western Japan) who spent their language formative years in the Kansai region (excluding Tajima in Hyogo Prefecture, the western area of Tango in Kyoto Prefecture, and Oku-Yoshino in Nara Prefecture).

[Exclusion criteria]

- Patients with problems in communication.
- Patients with hearing difficulties.
- Speakers of other dialects.
- Patients who are planning to start or make changes to their dementia treatment.

### Speech corpus

The study participants comprised 42 patients with dementia aged 69–98 years (mean: 84.95 years). We built a speech corpus based on the free narratives of these participants. To obtain the free narratives, we asked participants to provide responses to the following three questions:

- What is a recent happy event that you can remember?
- What is a happy past event in your life?
- What kinds of food do you like?

The narratives were recorded with a digital voice recorder and manually transcribed. The participants were also evaluated using the MMSE. See S1 File for the detailed characteristics of the participants.

## Computation of idea density in Japanese

ID was computed by calculating the number of propositions and dividing the sum by the number of tokens in the text. The methods for counting the number of tokens and the number of propositions are described below.

### Counting of tokens

Tokens were extracted using a Japanese morphological analyzer (Juman++) [8]. The analyzer extracted the following parts of speech based on the method proposed by Chand et al. [9]: the noun, verb, adverb, *i*-adjective (e.g., *aka-i*, “red”), *na*-adjective (e.g., *shizuka-na*, “quiet”), prenominals (e.g., *ikanaru*, “any”), conjunction, and emotive verb.

Note that we use the term “morpheme” to count tokens in Japanese. However, the term “word” is normally used to count tokens for English data because words in English sentences are generally separated by spaces; this facilitates the use of words as tokens. In contrast, words in normal Japanese writing are not segmented by spaces. In order to split Japanese sentences into tokens, we conducted segmentation of the sentences through the use of Japanese morphological analyzers such as Juman++ and MeCab [10]. In this paper, each output from the morphological analyzers was designated a “morpheme”, e.g., *Watashi*/*ha*/*akai*/*ringo*/*wo*/*nageta*. (“I threw a red apple.”). However, the definition of morpheme as an output from the Japanese morphological analyzers does not directly correspond to the notion of morpheme in linguistics; the former is slightly larger than the latter (e.g., the former segments *akai* as a single morpheme, whereas the latter segments this term into two morphemes of *aka*/*i*). In this way, our use of morpheme as an output from the morphological analyzers can be roughly regarded as corresponding to a word.

### Counting of ideas

The rules of annotation used in this study were, for the most part, based on the manual by Chand et al. [9]. According to that manual, ideas are composed of a predication, modification, and connectives. Fillers, laughter, unintelligible utterances, repetition, outsider’s utterances, and utterances directed to outsiders are not annotated. However, the rules developed for English cannot always be directly applied to Japanese on account of fundamental grammatical differences. Therefore, we added some new rules and modified some of the original rules to adapt this method for Japanese text. For detailed information on our rules, see S1 Appendix.

The number of ideas in the Japanese texts was manually counted by a linguist.

## Experiment 1: Computation of idea density using machine translation

The annotation of ideas in a text requires in-depth analysis by language experts, and therefore costs a substantial amount of time and money. Therefore, we propose a computational method to calculate ID automatically using machine translation from Japanese to English. This method employs automatic calculations of the number of ideas in machine-translated text based on procedures (DEPID) described in a previous study [5]. To verify the proposed method, we compared the number of ideas counted by human raters (J-ID) and those counted using DEPID (D-ID). In addition, we investigated the difference in the number of tokens between the original Japanese and translated English text, which can influence the calculation of ID.

### Methods

#### Ethics statement

The ethics committee of Kyoto University Hospital approved this research, including the provision of proxy consent (Approval No. C1152; April 28, 2016). Written informed consent was obtained from all participants and/or their respective proxies. Consent was obtained from a proxy when a patient lacked the autonomous competence to provide consent, in accordance with the Ministry of Health, Labour and Welfare’s Ethical Guidelines for Medical and Health Research Involving Human Subjects. Each participant’s autonomous competence was assessed by a physician with consideration to physician diagnosis and psychological examination (MMSE) by a psychologist before the interview. Participants were recruited between May and October 2016. We provided written information to all participants, which informed them about the discretionary nature of participation, the confidential treatment of data, and the possibility of withdrawing from the study.

#### Automatic computational method to calculate idea density in English

Two automatic ID computational methods have been previously developed for English texts. These are the CPIDR [4] and the DEPID [5] methods.

- CPIDR
  CPIDR is a tool for the automatic computing of ID from English text. This tool first processes the text using a part-of-speech tagger, then counts all verbs, adjectives, adverbs, prepositions, and coordinating conjunctions as propositions. It then applies a set of 37 rules to adjust the final proposition count. ID is computed by summing up the counts of propositions and normalizing them based on the number of word tokens.
- DEPID
  The DEPID method computes ID by calculating the counts of dependency relations and normalizing them based on the number of word tokens.

### Machine translation

As CPIDR and DEPID were designed for English text, we translated the Japanese text into English using three types of machine translation (Table 1). ID was then calculated from this English text using the DEPID method.

**Table 1:** Machine translations types.

Service	Method	Powered by
Google Translate	Neural Machine Translation (NMT)	Google
Bing Translator	Statistical Machine Translation (SMT)	Microsoft
Excite Translator	Phrase-Based Machine Translation (PBMT)	Excite Japan

In this study, ID was calculated based on dependency relations because Sirts et al. have reported that this method counts propositions more accurately than the CPIDR method [5].

DEPID was used to calculate ID (D-ID) by counting the number of propositions and dividing them by the number of tokens. The number of propositions (i.e., particular dependency relations in the case of D-ID) and number of tokens were counted based on the method used in the Stanford Parser [11].

In addition, the subject of a sentence is usually omitted from utterances in Japanese. This characteristic of the Japanese language can influence the counts of the tokens and calculation of ID. To clarify these differences, we compared the number of morphemes in the original Japanese corpus (calculated with a Japanese morphological analyzer) and the number of tokens in the translated corpus (calculated with the Stanford Parser).

$$\begin{array}{c} IdeaDensity=\frac{No.ofIdeas}{No.ofTokens} \end{array}$$ (1)

### Results

The number of ideas counted using DEPID was expected to be different from the number counted by the human raters. Therefore, we investigated their relationship by calculating the correlation coefficient and slope between these two variables.

**Fig 1:**
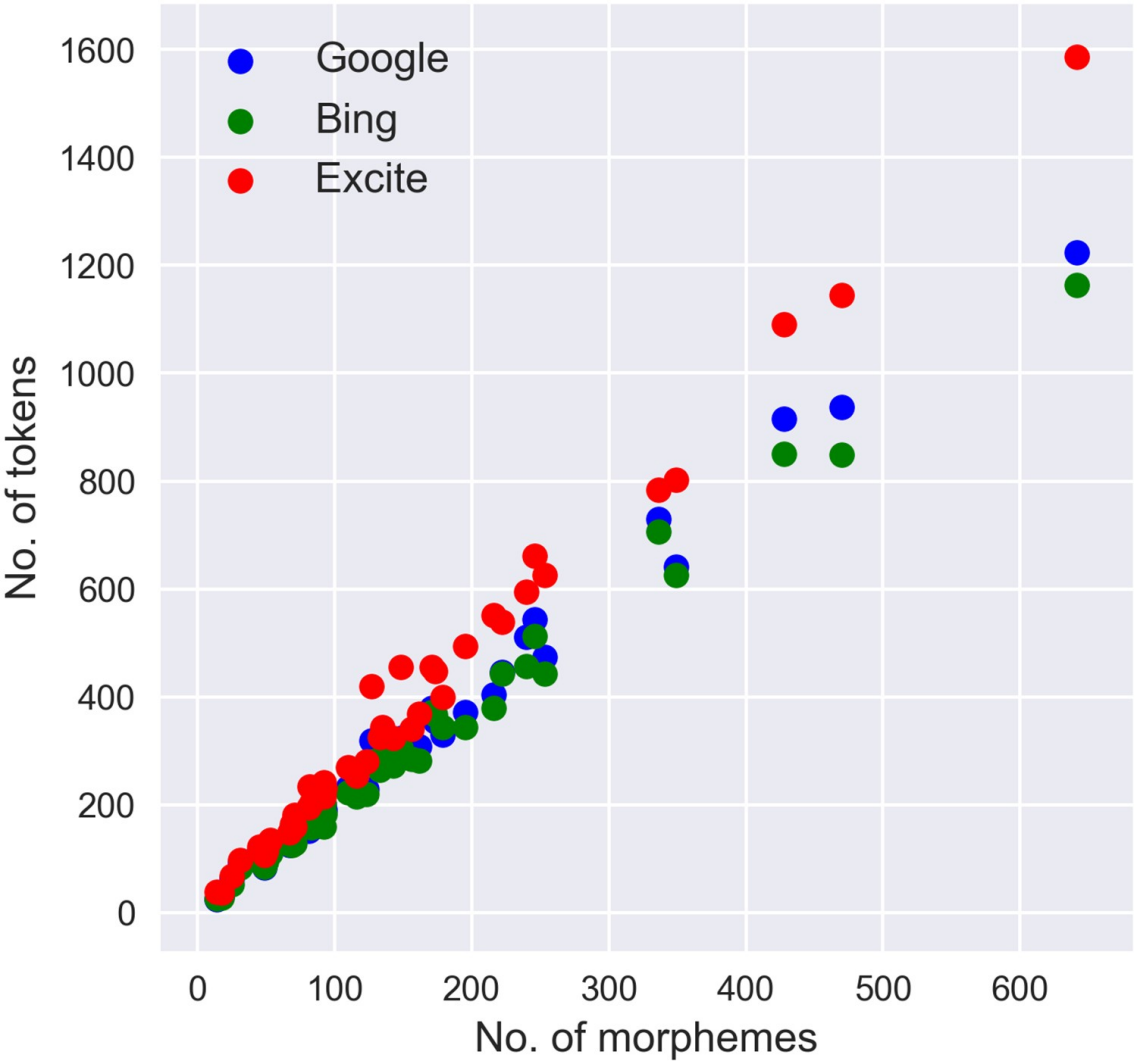
Correlation diagram between the number of morphemes and the number of tokens.

Table 2 presents the correlation coefficient and slope between the number of ideas counted using DEPID and the number of ideas counted by the human raters for the different machine translation types. The results showed that the highest correlation coefficient with the count by human raters was the DEPID count using Google Translate (*r* = 0.983), whereas the slope of the line yielded by DEPID using Bing Translator was the closest to 1 (*α* = 0.913). Fig 1 and Table 3 present the correlation coefficients and slopes between the number of morphemes and number of tokens. We confirmed that the correlation coefficient was constant (*r* = 0.995) in both the machine translations and slopes; the slope of the line using Bing Translator was the closest to 1 (*α* = 1.85).

**Table 2:** Spearman’s correlation and slope between the number of ideas in Japanese and English.

Method	Spearman’s *r*	Slope
Google	0.983	0.838
Bing	0.975	0.913
Excite	0.978	0.714

**Table 3:** Spearman’s correlation and slope between the number of morphemes and number of tokens.

Method	Spearman’s *r*	Slope
Google	0.995	1.97
Bing	0.995	1.85
Excite	0.995	2.46

### Discussion

With regard to the slope estimated using the results of Bing Translator, it was confirmed that there was an approximately 10% error (|observed value − true value|/ |true value|. In this case, the true value was equal to 1 because the numbers of ideas calculated by the two methods were the same.).

Although simple comparisons are inadequate, the results of our method using machine translations had approximately twice as many errors as that of a previous study [4]. However, the strong correlation between the numbers of ideas counted manually and automatically indicates that our estimation method has a fair degree of accuracy when considering its simplicity.

On the other hand, when considering the slope between the number of morphemes in the original Japanese text and the number of tokens in the text translated by Bing Translator, a morpheme in Japanese text was found to correspond to approximately 1.85 words in the translated English text. A possible reason for this finding is that the subject of a sentence is often omitted from utterances in Japanese during the manual count of ideas.

## Experiment 2: Application of idea density to dementia screening

With the aim of detecting dementia using ID, we calculated the correlation coefficient between ID and the MMSE score.

### Methods

**Fig 2:**
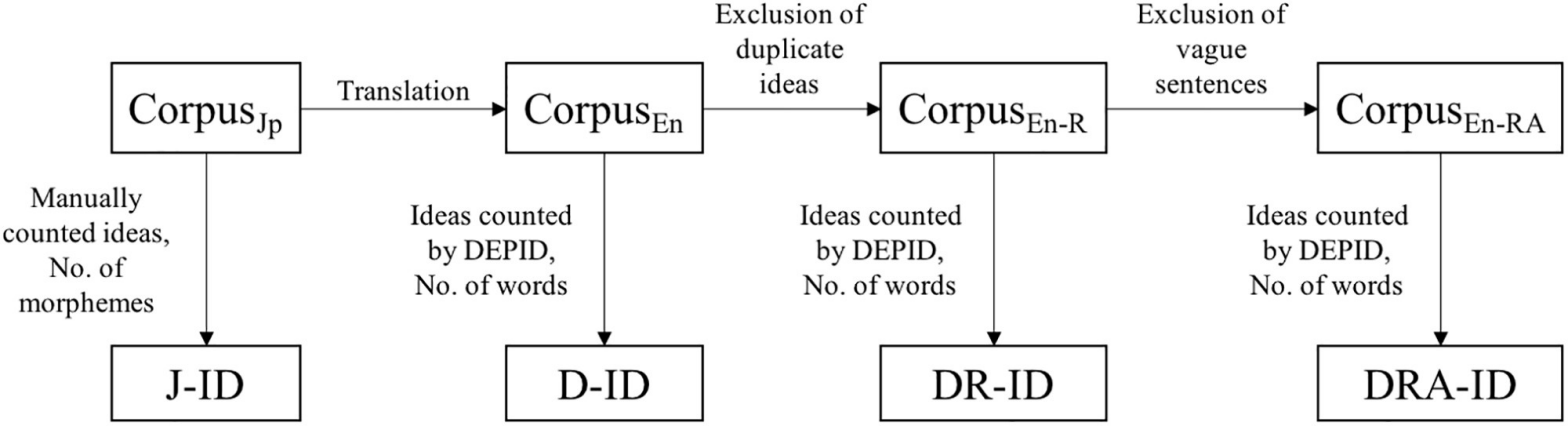
Procedure to calculate ID using various methods.

Sirts et al. [5] developed improved versions of DEPID called DEPID-R and DEPID-R-ADD, which are described below. We calculated ID using both DEPID-R (DR-ID) and DEPID-R-ADD (DRA-ID). Fig 2 summarizes the steps to calculate J-ID, D-ID, DR-ID, and DRA-ID from the original corpus.

- DEPID-R
  Sirts et al. [5] modified the original DEPID method to exclude duplicate ideas in a narrative by only counting proposition types. This modified version was designated DEPID-R. The difference between DEPID-R and DEPID is that the latter counts the tokens of the same propositions, whereas the former excludes repetitive counts. DR-Ideas indicates the number of ideas counted using DEPID-R.
  $$\begin{array}{c} IdeaDensity=\frac{No.ofDR-Ideas}{No.ofTokens} \end{array}$$ (2)
- DEPID-R-ADD
  DEPID-R-ADD is characterized by the addition of a function to exclude vague sentences through the use of the Speciteller tool [12]. One of the characteristics of dementia patients is the utterance of vague sentences that do not include specific ideas, such as the following examples:
  1. “The upper one is there.”
  2. “They’re doing more things on the outside.”
    DEPID-R-ADD calculates the concreteness of sentences. As suggested by Sirts et al., we did not count ideas from sentences with specificity scores below 0.01 [5]. DRA-Ideas indicates the number of ideas counted using DEPID-R-ADD.
    $$\begin{array}{c} IdeaDensity=\frac{No.ofDRA-Ideas}{No.ofTokens} \end{array}$$ (3)

We compared the following four values of ID: J-ID (calculated manually in Japanese), D-ID using DEPID, DR-ID using DEPID-R, and DRA-ID using DEPID-R-ADD.

### J-ID and MMSE

The correlation coefficient between J-ID and the MMSE score was −0.039, indicating no correlation between the two measures.

### D-ID and MMSE

The maximum correlation coefficient between D-ID and the MMSE score was 0.294 (Excite Translator), indicating a weak correlation (Table 4).

**Table 4:** Spearman’s correlation between D-ID and the MMSE score.

Method	Spearman’s *r*
Google	0.105
Bing	0.120
Excite	0.294

### DR-ID, DRA-ID, and the MMSE score

The maximum correlation coefficient between DR-ID and the MMSE score was 0.386 (Excite Translator), indicating a weak correlation. However, the maximum correlation coefficient between DRA-ID and the MMSE score was 0.473 (Google Translate), indicating a moderate correlation (Table 5).

**Table 5:** Spearman’s correlation between DR-ID, DRA-ID, and the MMSE score.

Version	Method	Spearman’s *r*
DEPID-R	Google	0.160
	Bing	0.060
	Excite	0.386
DEPID-R-ADD	Google	0.473
	Bing	0.334
	Excite	0.365

### Results

The correlation coefficient between J-ID and the MMSE score was −0.039; this result confirmed that the correlation between these measures was negligible. The correlation coefficients for D-ID and DR-ID with the MMSE score were 0.294 and 0.386, respectively (Excite Translator); these results confirmed weak correlations for these measures. The correlation coefficient between DRA-ID and the MMSE score was 0.473 for text translated using Google Translate, which indicated a moderate correlation between these measures.

### Comparison with baseline measures

Our analysis found a maximum correlation coefficient of 0.473 between DRA-ID and the MMSE score. To investigate the performance of DRA-ID, we counted the number of tokens (= the number of morphemes), nouns, verbs, adjectives, adverbs, adjectives and nouns, adverbs and verbs, and type-token ratio (TTR). We then calculated the correlation coefficients between the MMSE score and these eight linguistic measures. TTR is generally regarded to be a proxy for the vocabulary richness (or entropy) of a speaker, and is calculated by dividing the number of types by the number of tokens in a text. When counting the numbers of types and tokens, only content words (nouns, verbs, adjectives, adverbs) were counted. Table 6 shows the correlation coefficients between the MMSE score and the linguistic measures, as well as *p*-values for tests of non-correlation. Only the correlation between DRA-ID and the MMSE score was found to be statistically significant (*p*<0.05). As shown in Table 6, the correlation coefficients between the MMSE score and the eight linguistic measures were substantially lower than the correlation coefficient between the MMSE score and DRA-ID. Although it is difficult to compare the results of other linguistic measures from the same corpus, our measure was able to outperform baseline measures using a simple method.

**Table 6:** Correlation coefficients between the MMSE score and linguistic measures. Tests for non-correlation were used to calculate *p*-values. (*, *p*<0.05).

Measures	Correlation coefficient between MMSE score and linguistic measures	*p*-value
*DRA* − *ID*	0.473	0.002*
*Tokens*	0.153	0.330
*TTR*	0.021	0.900
*Nouns*	0.231	0.140
*Verbs*	0.062	0.700
*Adjectives*	0.126	0.430
*Adverbs*	0.253	0.110
*Adjectives and Nouns*	0.231	0.140
*Adverbs and Verbs*	0.062	0.700

### Discussion

The correlation coefficient between J-ID and the MMSE score was -0.039, which indicates a negligible correlation. However, D-ID, DR-ID, and DRA-ID demonstrated some degree of correlation with the MMSE score. The number of ideas counted from Japanese text using the DEPID method was generally consistent with the number counted by human raters.

Among the correlation coefficients examined, the highest coefficient was observed between DRA-ID (Google Translate) and the MMSE score at 0.473. In contrast, D-ID, which is the most similar method to J-ID, showed a low correlation coefficient with the MMSE score. This indicates that vague sentences represent noise from the viewpoint of detecting dementia. Sirts et al. [5] have also reported that vague sentences constitute noise in English text, and our results with machine translation corroborate their findings. Our results indicate that we may be able to apply methods designed for English text to Japanese text through the use of machine translations. ID detection for text (excluding vague sentences) appears to have evolved when compared to how it was originally defined by Kintsch and Keenan [3], but it represents a potentially effective tool for the early detection of dementia.

It is difficult to automatically calculate the number of ideas in Japanese because of the tendency to omit subjects in sentences. This sentence structure plays an important role in the counting of ideas. Sentences in English generally do not omit the subject, whereas sentences in Japanese frequently omit the subject, as shown in the following example:

(1) *Tamago-wo taberu*
 egg-OBJ eat-PRS
 ‘(I) eat an egg.’
 1. *taberu, (watashi-ga), tamago-wo*

However, the subject is often supplemented when a sentence is translated from Japanese into English by machine translation. Therefore, we regard our approach for calculating ID using machine translation as having the potential to be a fully automatic method.

Moreover, the differences among the translation engines may influence the performance of ID measurement. First, Excite Translator uses a rule-based machine translation engine, which parses the construction of a sentence and translates words using a dictionary. This results in a well-formed translation where the translated English sentences are likely to have subjects and objects even if they were omitted in the original Japanese. In addition, Excite Translator is thought to work better in translating professional writing than other methods as clearer terms can be chosen for translation instead of words with vague meanings; this may increase ID calculation performance. On the other hand, statistical and neural-based machine translation methods translate vague sentences as they are. These characteristics may have contributed to the poorer performance of D-ID and DR-ID using Google Translate and Bing Translator. After removing vague sentences (DRA-ID), these methods performed better than before.

Table 7 shows examples of translations provided by each machine translation system. First, all the systems returned appropriate translations for the relatively short sentence in Example (a). For the slightly longer and more informal Example (b), however, Google Translate returned a better translation than the other systems. In contrast, Bing Translator returned a grammatically correct but meaningless sentence, and Excite Translate returned an unintelligible sentence. As these examples show, Google Translate generally produced better results than the other systems. However, there were some exceptions that reflected an aspect of the nature of each machine translation system. Example (c) contains the term *funazushi* (crucian carp sushi), an uncommon word that was problematic for translation. In the result provided by Google Translate, the word was ignored and there was no equivalent. As demonstrated in this example, Google Translate would sometimes ignore unknown words. In contrast, the other two systems attempted to translate unknown words through word-by-word translation (Bing Translator) or transliteration (Excite Translator). Accordingly, we may conclude that Google Translate places a high priority on the fluency of the results, whereas the other two systems prioritize accuracy. In particular, the rule-based system used by Excite Translator appears to prefer word-by-word translation (sometimes transliteration). Bing Translator is based on statistical machine translation, which tends to provide a balance between the two methods. See the respective technical papers for further details of each translation system’s characteristics.

**Table 7:** Examples of translations provided by each machine translation system.

Original Japanese (transliterated to Latin characters)		Translated by a human	Google	Bing	Excite
(a)	*Eh chotto matte kudasai ne*.	Well, can you please wait a second?	Well, please wait a minute.	Oh, wait a minute.	Please wait a moment.
(b)	*Tabemono ha daitai nandemo tabe masu ne*.	I can eat nearly anything.	I do eat most of the food.	I usually eat food.	Everything almost eats food.
(c)	*Ano funazushi to ka kirai yo*.	I dislike that crucian carp sushi.	I mean, I hate it.	Carp, I hate sushi.	Uh, I don’t like crucian carp ZU SHI.

## Conclusion

In this paper, we proposed a method for estimating ID in Japanese narratives using machine translation and automatic idea counting.

We also reported the specifications of our method by calculating the correlation coefficients between ID and the MMSE score of patients with dementia. The maximum correlation coefficient was 0.473, which is indicative of a moderate correlation. This is the first study to calculate ID based on machine translation, and establishes the application of an automatic counting method for ideas in the Japanese language.

Here, we developed a method to count ideas in Japanese narratives. An accurate count can be obtained by first translating the target text into English using Bing Translator and then applying the DEPID method to the translated text. We also comparatively evaluated four ID calculation methods for the detection of dementia. The best method among those examined was the use of Google Translate to convert the Japanese text into English, which was then analyzed using DEPID-R-ADD.

While previous studies have compared ID between persons with dementia and healthy adults, we investigated the relationship between the language ability of a patient and his/her own stage of dementia. These findings suggest that our method can be applied to develop measuring tools to monitor the progression of this disease.

In this study, the specificity score threshold was set based on that used in a previous report. Therefore, there is a need to examine the effects of changes in this parameter to the results. In the future, we plan to investigate the repercussions of modifying this parameter and implement an end-to-end system to compare ID calculated from automatically and manually translated text in order to improve the robustness of our method.

## Supporting information

S1 File Minimal dataset for idea density and the Mini-Mental State Examination. Values over 89 in the “age” column were converted to “over 89” to ensure participant anonymity.
(XLSX)

S1 Appendix Rules for annotating idea density in Japanese text. (PDF)
