# Supplementary material for: Idea density in Japanese for the early detection of dementia based on narrative speech
Source: PLoS One. 2018 Dec 5;13(12):e0208418. doi: 10.1371/journal.pone.0208418 (PMC6281229; doi:10.1371/journal.pone.0208418)
Supplement: S1 Appendix — (PDF) [file pone.0208418.s002.pdf]

S1 Appendix: Rules for Annotating Idea Density to Japanese Text

Daisaku Shibata[1], Kaoru Ito[1], Hiroyuki Nagai[2], Taro Okahisa[2], Ayae Kinoshita[3], and Eiji Aramaki[1]

[1]Nara Institute of Science and Technology (NAIST), 8916–5 Takayama, Ikoma City, 630–0192, Japan
[2]Kyoto University Graduate School of Human and Environmental Studies, Yoshida-nihonmatsu-cho, Sakyo-ku, Kyoto City, 606-8501, Japan
[3]Kyoto University Graduate School of Medicine, 54 Kawahara-cho, Shogoin, Sakyo-ku, Kyoto City, 606–8507, Japan

July, 2018

Predication

The predicates in Japanese are verbs, nouns, i-adjectives, and na-adjectives. The predicate and its arguments were counted together as an idea (1). In line with [1], we did not count the markers of modality separately.

Verbs were counted together with their subjects and objects as ideas in the form *verb, subject, object*. Since subjects are often omitted in Japanese, we annotated verbs, supplementing their subjects if necessary. These supplemented subjects (e.g., the words in parentheses in (2)) were not counted as token numbers.

(1) Watashi-ga tamago-wo taberu
I-NOM egg-OBJ eat-PRS
'I eat an egg.'
1. *taberu, watashi-ga, tamago-wo*

(2) Tamago-wo taberu
egg-OBJ eat-PRS
'(I) eat an egg.'
1. *taberu, (watashi-ga), tamago-wo*

Subsidiary verbs were not counted as a separate idea. These were counted together with main verbs as one idea.

(3) Ani-ga gohan-wo tsukutte-kure-ta.
Brother-NOM meal-OBJ made-PASS-PST
'My brother made me a meal.'
1. *tsukutte-kure-ta, ani-ga, gohan-wo*

Nouns functioning as a predicate were treated as mentioned above(4). One-word sentences (5) counted as an idea.

(4) Watashi-wa isha da.
I-TOP doctor be-PRS
'I'm a doctor.'
1. *isha-da, watashi-wa*

(5) Osakana (as an answer to the question "What kinds of foods do you like?").
fish
'Fish'
1. *osakana*

i-adjectives and na-adjectives in predicative use were treated in the same way as verbs.

(6) Sono kuruma-wa aoi
that car-TOP blue-PRS
'The car is blue.'
1. *aoi, sono-kurumawa*

(7) Soko-wa shizukadatta
there-TOP quiet-PST
'There was quiet.'
1. *shizukadatta*, WHERE
2. WHERE = *soko*

Modification

Modification in Japanese includes i-adjectives, na-adjectives, adverbs, and adnominal modifiers. In addition to these, time and location nominals and negation were counted as an idea.

i-adjectives and na-adjectives in attributive use were counted separately.

(8) Aoi kuruma-wo katta
blue car-OBJ buy-PST
'(I) bought a blue car.'
1. *katta, (watashiga), kurumawo*
2. *kuruma, aoi*

(9) Shizukana kuruma-wo katta
silent car-OBJ buy-PST
'(I) bought a silent car.'
1. *katta, (watashiga), kurumawo*
2. *kuruma, shizukana*

Adverbs were counted as an idea.

(10) Yoku niku-wo taberu
often meat-OBJ eat-PRS
'(I) often eat meat.'
1. *taberu, (watashiga), nikuwo*
2. *taberu, yoku*

Time and location nominals were counted separately, adding "WHEN" and "WHERE."

(11) Kinou eiga-wo mita
yesterday movie-OBJ see-PST
'(I) saw a movie yesterday.'
1. *mita, (watashiga), eigawo*
2. WHEN = *kinou*

(12) gakkou-ni iku
school-LOC go-PRS
'(I) go to school.'
1. *iku, (watashiga)*, WHERE
2. WHERE = *gakkou*

When times or places were expressed by an embedded clause that had a restorable omitted subject, we also counted the ideas in that clause.

(13) Isoide gakkou-ni iku tochuu-de koronda
quickly school-LOC go halfway fall-PST
'(I) fell down on the way to school in a hurry.'
1. *iku, (watashiga)*, WHERE
2. WHERE = *gakkou*
3. *iku, isoide*
4. *koronda, (watashiga)*

Negatives were counted separately. The abbreviation "NEG" was used to show the presence of a negative.

(14) Watashi-ga tamago-wo tabe-nakatta
I-NOM egg-OBJ eat-NEG-PST
'I didn't eat an egg.'
1. *tabeta, watashiga, tamagowo*
2. NEG *nakatta*

Connectives

Connectives were counted as an independent idea. However, words used repeatedly in a narrative were not counted.

Conjunctions were counted as an independent idea.

(15) Ame-ga hutta kara komatta
rain-NOM fall-PST because have trouble-PST
'(I) was in trouble because it was raining. '
1. *hutta, amega*
2. *kara*
3. *komatta, (watashiga)*

(16) Gakkou-ni itta kedo yasumi datta
school-LOC go-PST but holiday be-PST
'(I) went to school, but it was a holiday. '
1. *itta, (watashiga)*, WHERE
2. WHERE = *gakkou*
3. *kedo*
4. *yasumidatta*

List of abbreviations

LOC	locative
NEG	negative
NOM	nominal
OBJ	object
PASS	passive
PRS	present
PST	past
TOP	topic

References

[1] Chand V, Baynes K, Bonnici LM, Farias ST. A rubric for extracting idea density from oral language samples. Current protocols in neuroscience. 2012; p. 10-5.
